# Musical Minds: Attentional Blink Reveals Modality-Specific Restrictions

**DOI:** 10.1371/journal.pone.0118294

**Published:** 2015-02-25

**Authors:** Sander Martens, Stefan M. Wierda, Mathijs Dun, Michal de Vries, Henderikus G. O. M. Smid

**Affiliations:** 1 Neuroimaging Center, University of Groningen, Groningen, the Netherlands; 2 Department of Neuroscience, University Medical Center Groningen, Groningen, the Netherlands; 3 Institute of Artificial Intelligence, University of Groningen, Groningen, the Netherlands; Goldsmiths, University of London, UK, UNITED KINGDOM

## Abstract

**Background:**

Formal musical training is known to have positive effects on attentional and executive functioning, processing speed, and working memory. Consequently, one may expect to find differences in the dynamics of temporal attention between musicians and non-musicians. Here we address the question whether that is indeed the case, and whether any beneficial effects of musical training on temporal attention are modality specific or generalize across sensory modalities.

**Methodology/Principal Findings:**

When two targets are presented in close temporal succession, most people fail to report the second target, a phenomenon known as the attentional blink (AB). We measured and compared AB magnitude for musicians and non-musicians using auditory or visually presented letters and digits. Relative to non-musicians, the auditory AB was both attenuated and delayed in musicians, whereas the visual AB was larger. Non-musicians with a large auditory AB tended to show a large visual AB. However, neither a positive nor negative correlation was found in musicians, suggesting that at least in musicians, attentional restrictions within each modality are completely separate.

**Conclusion/Significance:**

AB magnitude within one modality can generalize to another modality, but this turns out not to be the case for every individual. Formal musical training seems to have a domain-general, but modality-specific beneficial effect on selective attention. The results fit with the idea that a major source of attentional restriction as reflected in the AB lies in modality-specific, independent sensory systems rather than a central amodal system. The findings demonstrate that individual differences in AB magnitude can provide important information about the modular structure of human cognition.

## Introduction

“Music is the art of thinking with sounds”, according to the French music scholar Jules Combarieu (1859–1915). Being an important part of human culture, everyday exposure to music teaches most children basic musical competence, enabling them for instance to tap and dance to music, detect wrong notes, remember and reproduce familiar tunes and rhythms, and feel the emotions expressed in music [1]. On top of the common experience of listening to music, it has been shown that formal musical training can have a number of *domain-specific* effects on music perception [2–7] and can alter structure and function of localized brain areas [8–11]. Although there has been some controversy regarding the question whether formal musical experience can also have *domain-general* effects (i.e., effects that are not specific to the perception of music) [12], several studies have reported that explicit musical instruction benefits a range of domain-general abilities such as pre-reading and reading ability, mathematical and spatial abilities, creativity, and general intelligence [13–16].

Germane to the current study, beneficial effects on attentional and executive functioning, processing speed, and working memory have also been observed [17–20]. For instance, a recent study by Zuk et al. [20] found that musicians demonstrate enhanced performance on several constructs of cognitive flexibility, such as verbal fluency (the ability to name as many objects that match a certain criterion (e.g. animals) within 60 seconds), design fluency (the ability to connect a set series of dots to make as many different designs as possible within 60 seconds), and the Trail Making test (a measure of visual attention and task switching efficiency). In addition, they reported that musically trained children show increased brain activity in parietal areas and the right ventrolateral prefrontal cortex (VLPFC) when involved in rule representation and task-switching.

Interestingly, the same cognitive processes and associated brain areas have been suggested to play a crucial role in the Attentional Blink (AB) phenomenon [21]. The AB is a deficit in reporting the second of two targets when presented in close temporal succession. Being a central topic in attention research for more than two decades, it has proven to be a powerful tool to measure the temporal dynamics of attention [21–24]. As the AB can be obtained using a variety of stimuli and task conditions, it is thought to reflect a very general property of perceptual awareness [21]. However, while the AB is considered to arise from a fundamental restriction of attentional capacity and/or control, people differ widely in the magnitude of the AB, with some individuals (referred to as non-blinkers) showing little or even no AB [21,25–44]. When comparing these non-blinkers with strong blinkers, activation differences in parietal as well as the right VLPFC have been observed [25].

Given the spatial overlap of differential brain activity for non-blinkers (vs. blinkers) and musically trained (vs. untrained) individuals, it may be interesting to investigate the AB in musicians. If attentional and executive functioning, processing speed, and working memory are indeed enhanced in musicians, one would predict that they may also show a reduced AB effect, similar to what has also been observed in expert meditators [38] and video gamers [40]. Therefore, the primary goal of the current study was to determine whether the beneficial effect of musical training on attention and working memory is reflected in the AB.

If, in comparison to non-musicians, an effect can indeed be observed, an intriguing subsequent question will be whether it is present within the auditory modality only, or whether the positive effect of musical training on the efficiency of selective attention carries over to both the auditory and the visual modality. Both cases would provide evidence for a *domain-general* effect of musical training experience on selective attention. In contrast, a *modality-specific* effect (i.e., auditory only) would pinpoint boundaries in and between attention and ‘the art of thinking in sounds’. To test these hypotheses, two experiments were carried out using stimuli of equivalent difficulty that were either presented in the auditory (Experiment 1) or visual modality (Experiment 2). In each experiment, a group of musicians and a group of non-musicians performed an AB task, requiring the identification of letter targets amongst a rapid sequential stream of digit distractors.

## Methods

### Experiment 1

Participants

A group of 29 volunteers (aged 18–34, mean = 22.4 years) formed the musicians group, recruited from the University of Groningen community and the Prince Claus Conservatoire. All musicians had attended lessons in playing one or more musical instruments for at least 4 years and actively played a minimum of 4 hours a week. Twenty-nine additional volunteers, recruited from the University of Groningen community (aged 18–33, mean = 22.2), formed the control group (“non-musicians”). Unlike the musicians, they reported to have no noteworthy musical background.

All participants had Dutch as their native language, normal or corrected-to-normal visual acuity, normal hearing and no history of neurological problems. The Neuroimaging Center Institutional Review Board approved the experimental protocol and written consent was obtained prior to the experiment. Participants received payment of € 6.

Stimuli and Apparatus

Stimuli consisted of spoken consonant letters (excluding ‘S’ and ‘V’) and digits (excluding ‘1’, ‘5’, ‘6’, ‘7’, and ‘9’), which were digitally recorded and compressed to 120 ms duration. The stimuli were presented at approximately 83 dB using Sony MDR-V600 headphones. The generation of stimuli and the collection of responses were controlled by using E-prime 1.2 software [45] running under Windows XP on a PC with a 2.8-GHz processor.

Procedure

Prior to each trial, a fixation point appeared in the middle of a 17-inch CRT monitor together with a message at the bottom, prompting participants to press the space bar to initiate the trial. When the space bar was pressed, the message disappeared immediately. The fixation cross remained on the screen for 250 ms, followed by a sequential stream of 22 stimuli. Each item was presented for 120 ms, with an inter stimulus interval of 10 ms. In 80% of the trials, two target letters were embedded in the stream (dual-target trials), and in 20% of the trials, only one target was present (single-target trials). In single- and dual-target trials, T1 was always presented as the fifth item in the stream. In dual-target trials, T2 was the first, second, third, or twelfth item following T1 (i.e., T2 was presented at lag 1, 2, 3, or 12, respectively). Thus, the stimulus onset asynchrony (SOA) between the targets randomly varied from 130, 260, 390, to 1560 ms. Each SOA was presented equally often. Target letters were randomly selected with the constraint that T1 and T2 were always different letters. Digit distractors were randomly selected with the constraint that no single digit was presented twice in succession. After the presentation of the stimulus stream, participants were asked to identify the presented targets, if possible, by pressing the corresponding keys on the computer keyboard. Participants were instructed to take sufficient time in making their responses to ensure that typing errors were not made. If a target was missed or absent, participants were instructed to press the space bar instead. Responses were accepted and counted correct in either order. After responses were collected, participants could initiate the next trial by pressing the space bar.

The experiment consisted of three practice blocks and two experimental blocks. In the first practice block, all 23 stimuli were presented one by one, in isolation. Participants identified each stimulus by pressing the corresponding key on a keyboard. When all stimuli had been presented once, stimuli that were not correctly identified were presented again in random order, until all stimuli were identified correctly. The second practice block contained 24 single-target trials, during which participants were required to identify the single target letter embedded within the stream of digit distractors. Feedback was provided at the end of each trial for 1 s. The block was repeated as long as accuracy remained below 70% (once for n = 15, twice for n = 3, three times for n = 3). In the last practice block as well as in the experimental blocks, stimulus streams with either 1 or 2 targets were presented, as described above. The practice block consisted of 30 trials, whereas the two experimental blocks consisted of 160 trials each. Feedback was provided in the practice block only. After the first experimental block, participants were allowed to take a short break. The experiment took approximately 45 minutes to complete.

### Experiment 2

To investigate whether a reduced auditory AB is accompanied by a reduced visual AB, an identification task similar to the auditory AB task was given, but using visual stimuli, similar to [34,35].

Participants

All participants from Experiment 1 were re-invited and volunteered to participate in Experiment 2. The Neuroimaging Center Institutional Review Board approved the experimental protocol and written consent was obtained prior to the experiment. Participants received payment of € 4.

Stimuli and Apparatus

Stimuli consisted of consonant letters (excluding ‘Q’) and digits (excluding ‘0’ and ‘1’). They were presented in black on a white background presented in 12-point Courier New font, using the same hard- and software as in Experiment 1.

Procedure

Prior to each trial, a fixation cross was presented in the middle of the screen. When the space bar was pressed, the fixation cross disappeared immediately and was followed 100 ms later by the rapid serial visual presentation (RSVP) stream. Like the auditory stimulus stream in Experiment 1, the visual stream consisted of 22 stimuli, containing one target in 20% of the trials, and two targets in 80% of the trials.

In contrast to Experiment 1, distractors in the visual AB task were presented for 90 ms. Following [32,34–36], we attempted to control task difficulty, keeping mean visual T1 performance in Experiment 2 equivalent to the mean auditory T1 performance in Experiment 1, for each individual participant, by manipulating the duration of visual targets in the following way.

Each block of trials began with a target duration of 70 ms, immediately followed by a 20-ms mask (a digit). After the first trial, target and mask duration were variable, with target duration ranging from 20 to 80 ms. The sum of target and mask duration was always 90 ms, thereby keeping the interval between the onset of a target and the onset of a subsequent distractor constant. After each trial, a running average of T1 accuracy was calculated and compared to that individual’s mean T1 accuracy in Experiment 1. Whenever mean T1 accuracy in Experiment 2 became 5% higher than the mean T1 accuracy in Experiment 1, visual target presentation duration was decreased by 10 ms and mask duration was increased by 10 ms, thereby making visual target identification more difficult. When mean visual T1 accuracy became 5% lower than the mean auditory T1 accuracy (in Experiment 1), visual target duration was increased by 10 ms and mask duration decreased by 10 ms, thereby making visual target identification easier. The identity of a mask never corresponded with the identity of a preceding or following distractor digit.

The first target was always presented as the fifth item in the stream. T2 was the first, second, third, or twelfth item following T1 (i.e., it was presented at lag 1, 2, 3, or 12, respectively), resulting in SOAs of 90, 180, 270, and 1080 ms. Note that lag 2 in the auditory experiment corresponded roughly with lag 3 in the visual experiment (SOAs of 260 and 270, respectively). Response instructions as in Experiment 1 were given.

The experiment consisted of one practice block of 30 trials and two experimental blocks of 160 trials each. After each block, participants were allowed to take a short break. The experiment took approximately 30 minutes to complete.

## Results and Discussion

### Experiment 1

**Fig 1:**
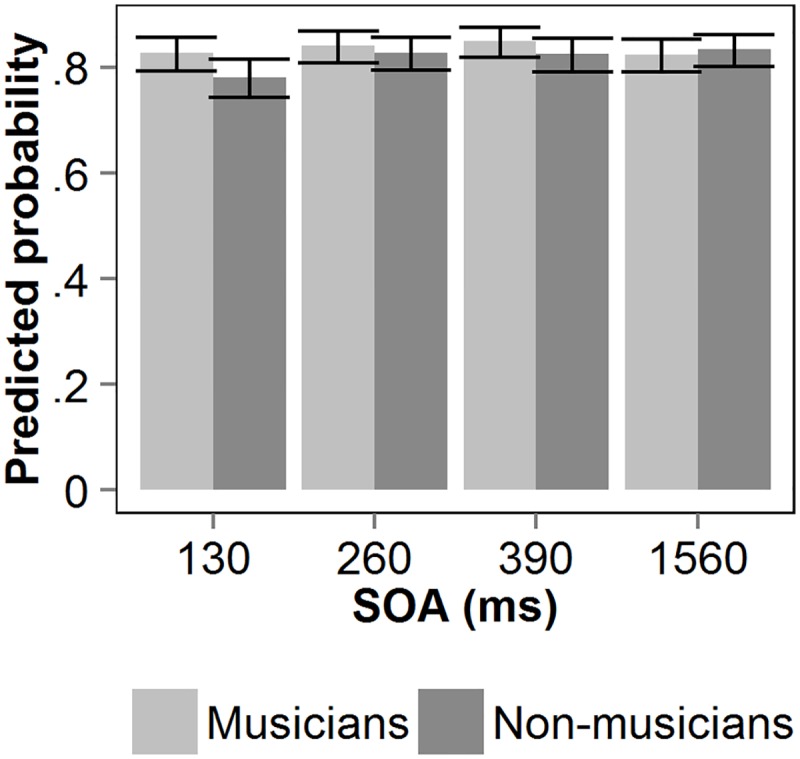
Auditory T1 performance in Experiment 1. Predicted probability of correct T1 identification as a function of stimulus onset asynchrony (SOA) between the two auditory targets, for non-musicians and musicians. Error bars reflect upper and lower confidence limits of the model.

Following [46,47], the results were analyzed using binomial mixed-effects models. Given that there are large individual differences in the AB [25] and that our hypothesis predicted a different number of observations per cell, mixed-effects models are preferred over methods that assume an equal number of observations per cell. The use of mixed-effects models in the field of AB research is relatively new, but the method is widely used in other fields, such as psycholinguistics [48], eye movement data [49], or memory research [50]. Analyses were performed using lmer functions in the lme4 package [51] for the statistical software R. Fig. 1 shows the predicted probability of correct T1 identification as a function of the interval between the two targets (SOA) for both groups of participants. Note that the probabilities that are depicted in the figures were predicted by the mixed-effects models, which were fit to the binomial accuracy data. Also, the error bars show the upper and lower confidence limits as estimated by the model.

A binominal mixed-effects model was fitted on T1 accuracy. SOA and group were entered as fixed factors in the model. Subject was entered as a random factor. The model explains significantly more variance compared to the null-model, χ^2^(7) = 28.014, *p* <. 001. The model’s estimates, standard errors, *z*-values and *p*-values are listed in Table 1. Performance of the non-musicians at a SOA of 1560 ms (i.e., lag 12) was taken as baseline. The negative estimate of SOA 130 (i.e., lag 1), reflects interference between the two targets at the first lag. In addition, there was a significant Group × SOA interaction at SOA 130 and a marginally significant Group × SOA interaction at SOA 390. The direction and size of the estimates indicate less interference between the targets at these SOAs for the musicians compared to the non-musicians. No significant difference (*p* = .2) in single target performance was found between musicians (84.0%) and non-musicians (82.0%).

**Table 1:** The estimates and z-values of the mixed-effects model for T1 accuracy of the auditory AB experiment.

	Mixed-effects model T1			
	Estimate	Standard Error	z-value	p-value
Non-musicians, SOA 1560(intercept)	1.615	0.112	14.404	0.000
Musicians	-0.066	0.158	-0.415	0.678
SOA 130	-0.344	0.084	-4.112	0.000
SOA 260	-0.042	0.087	-0.479	0.632
SOA 390	-0.060	0.087	-0.695	0.487
Musicians, SOA 130	0.359	0.120	2.993	0.003
Musicians, SOA 260	0.156	0.123	1.265	0.206
Musicians, SOA 390	0.238	0.124	1.928	0.054

The estimates of the fixed effects are given in log odds.

**Fig 2:**
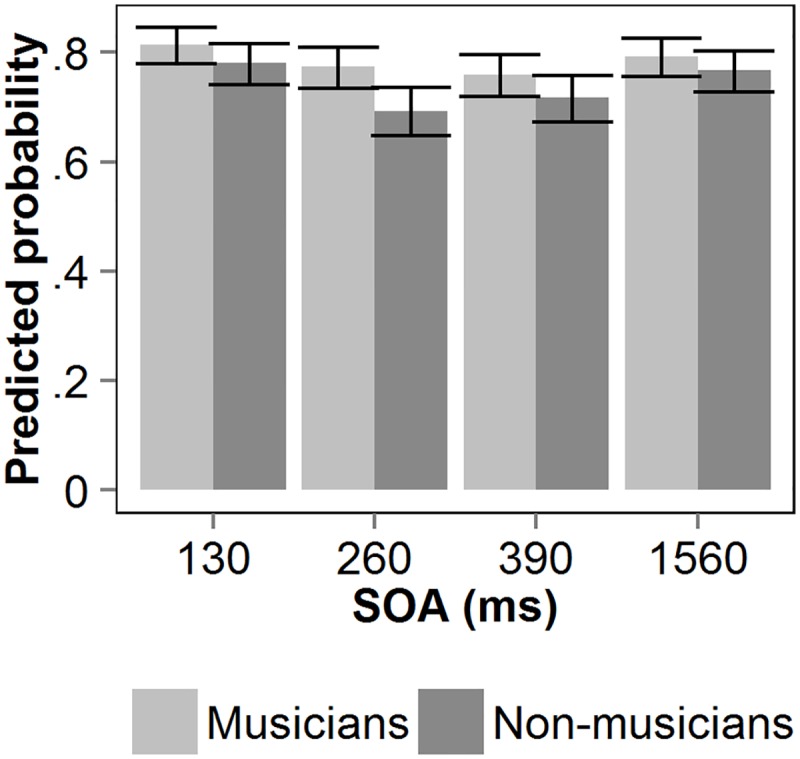
Auditory T2|T1 performance in Experiment 1. Predicted probability of T2 given correct report of T1, as a function of stimulus onset asynchrony (SOA) between the two auditory targets, for non-musicians and musicians. Error bars reflect upper and lower confidence limits of the model.

Fig. 2 shows the predicted probability of T2 given a correct T1 (T2|T1) as a function of the interval between the two targets (SOA) for both groups of participants. A binomial mixed-effects model was fitted on the accuracy of T2 given correct report of T1. The factors SOA and group were entered in the model as fixed factors. Subject was entered as random factor. The model explains significantly more variance compared to the null-model, χ^2^(7) = 56.676, *p* <. 001. Table 2 shows the model’s estimates, standard errors, *z*-values and *p*-values. The model revealed an effect of SOA at 260 and 390 ms. The negative estimates at both SOAs reflect the occurrence of an AB. A significant Group × SOA interaction was found for SOA 260. The positive estimate indicates that musicians performed better at this SOA compared to the non-musicians. A separate analysis of the musicians’ performance only revealed a significant effect of SOA at 390 ms (*z* = -2.19, *p* = .03). In other words, both groups showed a significant AB at SOA 390. Importantly, however, the musicians’ absence of an AB at SOA 260, combined with the fact that their AB at SOA 390 was not as large as that of the non-musicians at SOA 260, suggest that the auditory AB in musicians was both delayed *and* attenuated.

**Table 2:** The estimates and z-values of the mixed-effects model for T2|T1 accuracy of the auditory AB experiment.

	Mixed-effects model T2|T1			
	Estimate	Standard Error	z-value	p-value
Non-musicians, SOA 1560(intercept)	1.191	0.109	10.981	0.000
Musicians	0.151	0.155	0.973	0.330
SOA 130	0.077	0.089	0.866	0.386
SOA 260	-0.377	0.084	-4.514	0.000
SOA 390	-0.263	0.084	-3.124	0.002
Musicians, SOA 130	0.059	0.129	0.457	0.648
Musicians, SOA 260	0.267	0.122	2.181	0.029
Musicians, SOA 390	0.070	0.577	2.850	0.564

The estimates of the fixed effects are given in log odds.

### Experiment 2

**Fig 3:**
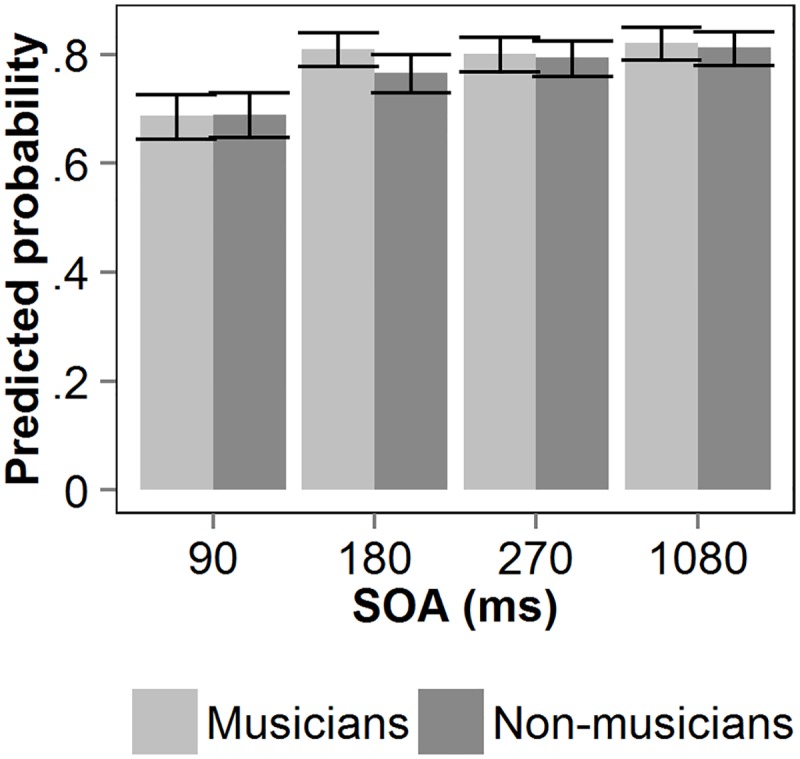
Visual T1 performance in Experiment 2. Predicted probability of correct T1 identification as a function of stimulus onset asynchrony (SOA) between the two visual targets, for non-musicians and musicians. Error bars reflect upper and lower confidence limits of the model.

Fig. 3 shows the predicted probability of correct T1 identification as a function of the interval between the two targets (SOA) for both groups of participants.

A binominal mixed-effects model was fitted on T1 accuracy. SOA and group were entered as fixed factors in the model. Subject was entered as a random factor. The model explains significantly more variance compared to the null-model, χ^2^(7) = 206.23, *p* <. 001. The model’s estimates, standard errors, *z*-values and *p*-values are listed in Table 3. The effect found for SOAs 90 and 180 reflects interference between the two targets. The lack of a significant main group effect or interaction indicates that T1 performance was equivalent for the two groups.

**Table 3:** The estimates and z-values of the mixed-effects model for T1 accuracy of the visual AB experiment.

	Mixed-effects model T1			
	Estimate	Standard Error	z-value	p-value
Non-musicians, SOA 1080(intercept)	1.464	0.102	14.303	0.000
Musicians	0.064	0.145	0.437	0.662
SOA 90	-0.667	0.078	-8.528	0.000
SOA 180	-0.278	0.081	-3.434	0.001
SOA 270	-0.113	0.083	-1.367	0.172
Musicians, SOA 90	-0.074	0.111	-0.667	0.505
Musicians, SOA 180	0.200	0.117	1.712	0.087
Musicians, SOA 270	-0.020	0.118	-0.171	0.864

The estimates of the fixed effects are given in log odds.

No significant difference (*p* = .7) in single target performance was found between musicians (77.1%) and non-musicians (75.8%). Using a mixed-effect model to analyze mean target duration, calculating the *p-*value by performing 10000 Markov Chain Monte Carlo (MCMC) samplings, no significant difference was found between musicians (71 ms) and non-musicians (72 ms), *t* = .21, *p* = .83. A comparison between (dual-target) T1 performance in Experiment 1 and 2 revealed that mean auditory T1 performance (80.3%) was significantly better (*z* = -7.45, *p* <. 001) than the mean visual T1 performance (76.4%), but that these differences were similar for both groups (*p*s >. 3).

**Fig 4:**
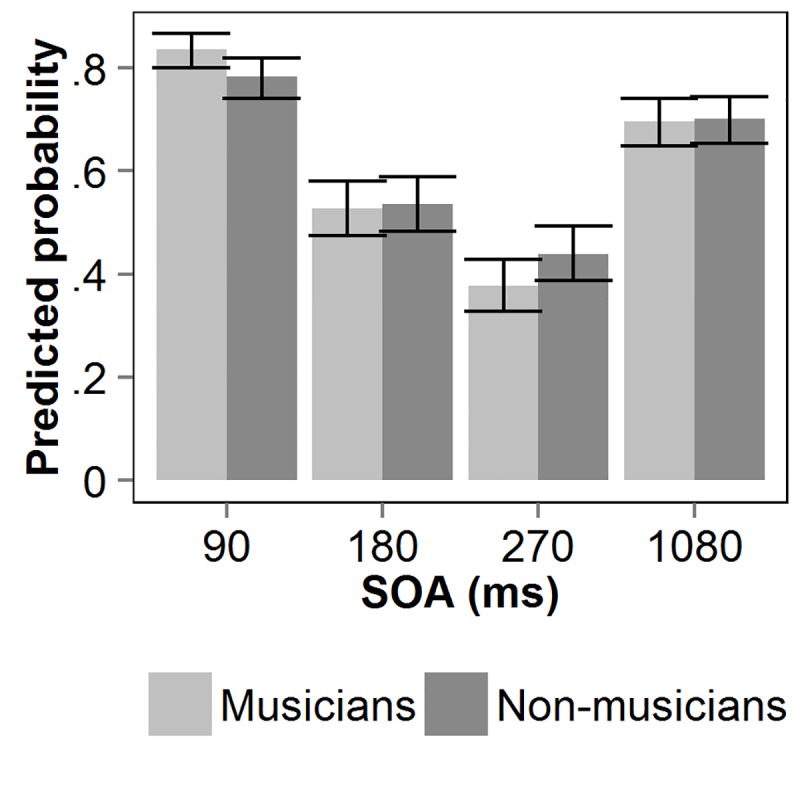
Visual T2|T1 performance in Experiment 2. Predicted probability of T2 given correct report of T1, as a function of stimulus onset asynchrony (SOA) between the two visual targets, for non-musicians and musicians. Error bars reflect upper and lower confidence limits of the model.

Fig. 4 shows the predicted probability of T2 given a correct T1 (T2|T1) as a function of the interval between the two targets (SOA) for both groups of participants. A binomial mixed-effects model was fitted on the accuracy of T2 given correct report of T1. The factors SOA and group were entered in the model as fixed factors. Subject was entered as random factor. The model explains significantly more variance compared to the null-model, χ^2^(7) = 1089, *p* <. 001. Table 4 shows the model’s estimates, standard errors, *z*-values and *p*-values. The model revealed an effect of SOA at 90, 180, and 270 ms. The negative estimates at SOAs 180 and 270 reflect the occurrence of an AB, whereas the positive estimate at SOA 90 reflects lag-1 sparing [52]. Although there was no main effect of group, somewhat surprisingly, a significant Group × SOA interaction was found for SOAs 90 and 270. The positive estimate at the SOA of 90 ms indicates stronger lag-1 sparing for musicians, whereas the negative estimate at the SOA of 270 ms reflects a greater AB magnitude for musicians than for non-musicians.

**Table 4:** The estimates and z-values of the mixed-effects model for T2|T1 accuracy of the visual AB experiment.

	Mixed-effects model T2|T1			
	Estimate	Standard Error	z-value	p-value
Non-musicians, SOA 1080(intercept)	0.850	0.111	7.658	0.000
Musicians	-0.02170	0.157	-0.138	0.899
SOA 90	0.432	0.090	4.786	0.000
SOA 180	-0.703	0.080	-8.785	0.000
SOA 270	-1.094	0.080	-13.735	0.000
Musicians, SOA 90	0.372	0.131	2.829	0.005
Musicians, SOA 180	-0.017	0.112	-0.154	0.878
Musicians, SOA 270	-0.236	0.113	-2.099	0.036

The estimates of the fixed effects are given in log odds.

**Fig 5:**
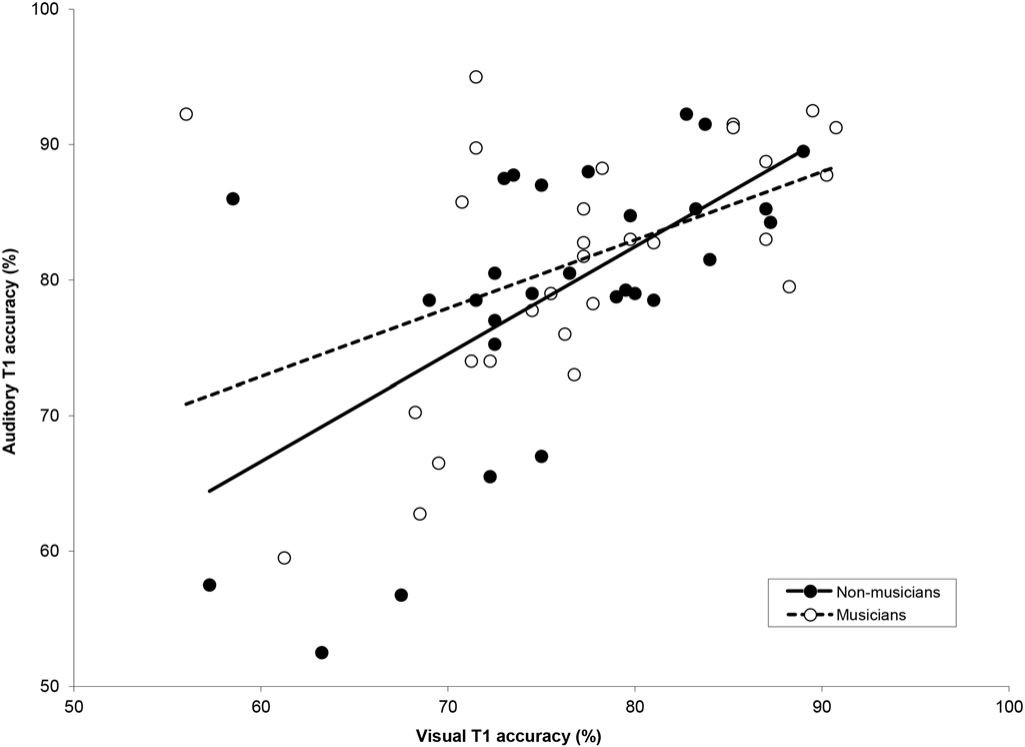
Mean T1 accuracy in Experiment 1 and 2. The correlation between individual T1 accuracy in the visual modality (Experiment 2) versus auditory modality (Experiment 1).

**Fig 6:**
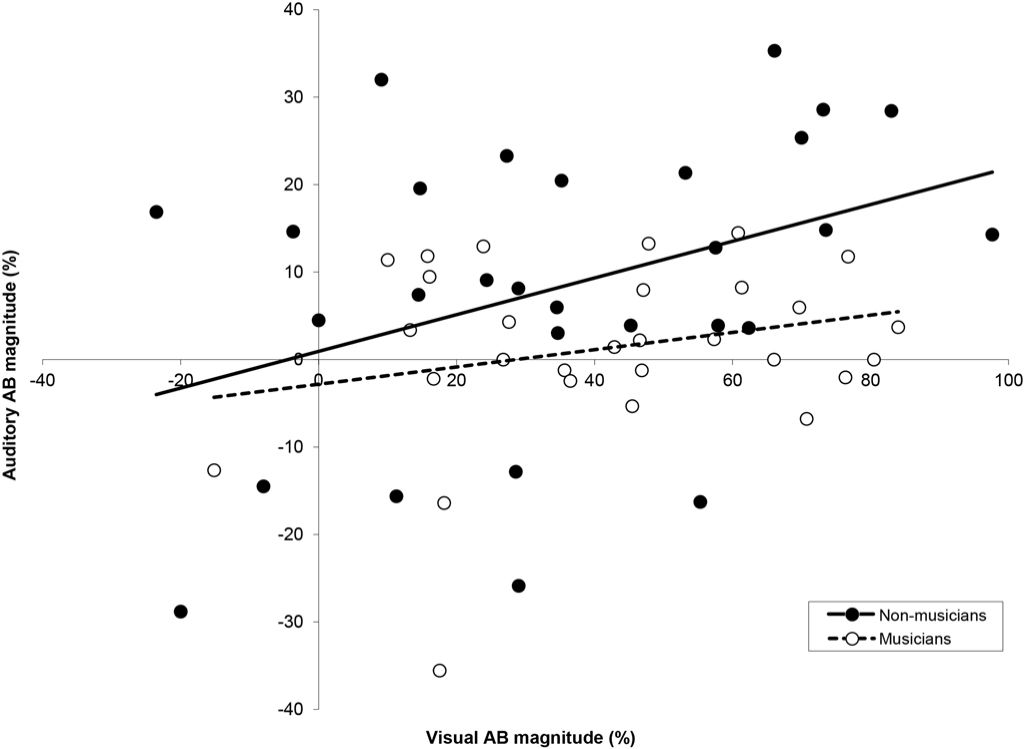
AB magnitudes in Experiment 1 and 2. The correlation between individual AB magnitudes in the visual modality (Experiment 2) versus auditory modality (Experiment 1). Large visual blinkers tend to be large auditory blinkers, unless one is a musician.

When mean individual T1 performance within the auditory modality was compared to that within the visual modality as shown in Fig. 5, significant positive Pearson product-moment correlations were found for each group (non-musicians: *r* = .61, *p* <. 001; musicians: *r* = .47, *p* = .01). In an additional analysis shown in Fig. 6, we correlated auditory AB magnitude with visual AB magnitude, for musicians and non-musicians respectively. Auditory AB magnitude was calculated using the following formula:

$$\left(\frac{T2|T1_{SOA1560}-T2|T1_{SOA260}}{T2|T1_{SOA1560}}\right)*100\%$$ (1)

In other words, auditory AB magnitude reflected the decrement in performance at SOA 260 relative to the longest SOA. Visual AB magnitude was calculated similarly, based on SOAs 270 and 1080. For non-musicians, a significant positive Pearson product-moment correlation was found, *r* = .39, *p* = .04, indicating that non-musicians with a relatively large auditory AB also tend to show a large visual AB. For musicians, however, no significant correlation between auditory and visual AB magnitude was found (*p* = .21). A similar pattern of results was found when the difference in performance between the long and short SOA was taken as a measure of AB magnitude. The lack of a significant (negative) correlation in musicians suggests that there is no simple trade-off between modality-specific attentional capacity or strategy.

## General Discussion

Even though the AB has long been considered as a fundamental bottleneck to become consciously aware of relevant information, several recent studies have argued against the existence of a hard-wired limitation, showing that experimental manipulations can strongly affect AB magnitude [38–40,46,53–62]. In addition, large individual differences in AB magnitude have been reported (e.g., [25]). If temporal attention is indeed under strategic control that varies between individuals, an important question is how general such a processing strategy is within individuals.

To our knowledge, this is the first study to show that the auditory AB in musicians is both delayed and attenuated using non-musical stimuli, possibly reflecting a more efficient attentional distribution [25,39,44,46,47,54,63]. In contrast, the distribution of attention within the visual modality seems less optimal, evidenced by a larger visual AB and more lag-1 sparing in musicians, relative to performance in participants without musical background. The domain-general but modality-specific effect of formal musical training is congruent with a study on mental imagery, which reported that musicians outperformed non-musicians on a non-musical auditory imagery task, but not on a visual imagery task [8].

In line with previous results [35], non-musicians with a large auditory AB also showed a large visual AB, suggesting that a similar target selection strategy was used for both modalities. Indeed individual auditory T1 performance correlated with visual T1 performance, for both groups. However, neither a positive nor a negative correlation was found between the musicians’ auditory and visual AB magnitudes, suggesting that at least in musicians, attentional restrictions within each modality are completely separate.

Whereas we previously reported that some individuals (non-blinkers) can show a significant auditory AB but little or no visual AB [34], the present study demonstrates that the opposite result can also be found: A given individual can show substantial attentional restrictions within the visual modality but reduced restrictions within the auditory modality. This contrasting pattern of results provides further evidence that a major source of attentional restriction as reflected in the AB lies in modality-specific, independent sensory systems rather than a central amodal system.

While it must be noted that attentional selection of multiple targets is likely to be restricted not only by modality-specific but also by central limitations [64–68], our current findings suggest that musical training seems particularly beneficial within the auditory modality rather than at a central amodal level. The particular expertise that musicians have gained through years of training may have optimized the formation as well as handling of auditory representations. This may allow for a more optimal timing of attentional allocation to auditory targets [58] similar to the more optimal deployment of attention for visual targets that has been observed in non-blinkers [25]. Though we did not find a negative correlation between visual and auditory AB magnitudes in musicians, their relative expertise for auditory information may have caused them to put relatively more effort in the identification of visual targets. The musicians’ increased lag-1 sparing as well as AB magnitude in our visual AB task provides support for this idea, in line with the overinvestment hypothesis that we and others have previously suggested [46,47,53,63].

Although we find it plausible to assume that musical training caused the described changes in attentional restrictions, additional research is required in order to empirically validate this assumption. If musical training really causes a decrease in auditory AB magnitude, it would be interesting to determine which aspects of the training actually contribute to the effect on the one hand, and which specific cognitive processes that are known to play a role in the AB are affected on the other hand.

## Conclusions

We conclude that people who are musically trained show an attenuated and delayed AB when required to identify two auditory targets amongst a stream of non-targets, possibly reflecting a more efficient allocation of attention. In contrast, the distribution of attention within the visual modality seems less optimal, evidenced by a larger visual AB and more lag-1 sparing in musicians, relative to performance in participants without musical background.

Although in the current study non-musicians with a relatively large auditory AB also tend to show a relatively large visual AB (also see [35]), neither a positive nor negative correlation was found for the musicians. The results reported here fit with the idea that a major source of attentional restriction as reflected in the AB lies in modality-specific, independent sensory systems rather than a central amodal system [35,65,69,70]. This does not mean that attention is not limited at a central level. For instance, the positive correlation between visual and auditory T1 accuracy that we generally observed in both musicians and non-musicians may well arise from individual restrictions at a more central, amodal level.

In addition, the results show that formal musical training can indeed have domain-general (i.e., not specific to the perception of music) beneficial effects, but that at least some of these effects are limited to the auditory modality and do not carry over to the visual modality. Taken together, the findings demonstrate that individual differences in AB magnitude can provide important information about the modular structure of human cognition.

## Supporting Information

S1 Dataset (CSV)
